# Isopropanol sensor based on sprayed In_2_S_3_ thin film using linear discriminant analysis for real-time selectivity

**DOI:** 10.1039/d4ra03498h

**Published:** 2024-07-26

**Authors:** R. Souissi, B. Bouricha, N. Ihzaz, N. Bouguila, M. Abderrabba

**Affiliations:** a Laboratory of Materials, Molecules and Applications IPEST, University of Carthage BP 51 La Marsa 2070 Tunis Tunisia riadhsouissi1@gmail.com +216 28419444; b Preparatory Institute of Engineering Studies of Bizerte, University of Carthage Zarzouna 7021 Bizerte Tunisia; c Faculty of Sciences of Bizerte, University of Carthage Zarzouna 7021 Bizerte Tunisia; d Faculty of Sciences of Gabes, Laboratory of Physics of Materials and Nanomaterials Applied to the Environment, University of Gabes Erriadh 6079 Gabes Tunisia

## Abstract

Metal sulfides have been studied for their high performance as new sensitive materials for gas detection. These material innovations contribute significantly to the development of more sensitive, stable and specific conductivity sensors, opening the way to new applications in detecting gases at low concentrations. Hence, this work reports on the sensing performance of In_2_S_3_ for isopropanol detection. Numerous VOCs, such as ketones, alcohols, and aldehydes, serve as cancer biomarkers. Notably, isopropanol, as a biomarker, shows a substantial increase (20–1007 ppb) in lung cancer patients, suggesting its potential as an early diagnostic criterion. To explore this, we fabricated a thin film of In_2_S_3_ onto a platinum interdigitated silicon dioxide substrate by simple and low-cost spray pyrolysis technology. Structural and morphological analyses *via* XRD, MEB, AFM, and TEM were conducted on the prepared samples. Isopropanol vapor response was assessed within a controlled temperature range of 250 °C–450 °C. The In_2_S_3_-based sensor demonstrated notable sensitivity (0.034 ppm^−1^), maintained stability over three weeks, and reliably detected isopropanol activation. In_2_S_3_ emerges as a promising candidate for detecting isopropanol, with a limit of detection (LOD) of 162 ppb. In this work, seven VOCs, including isopropanol, ethanol, methanol, butan-1-ol, formaldehyde, toluene, and acetone, were evaluated. Cross-responses among these VOCs were observed, indicating a lack of assured sensor selectivity. However, isopropanol recognition was achieved by employing linear discriminant analysis (LDA) on pertinent features derived from transient current change measurements. As a result, the sensor's sensitivity enables the deduction of the isopropanol concentration.

## Abstract 2

Metal sulfides have been studied for their high performance as new sensitive materials for gas detection.

## Graphical abstract

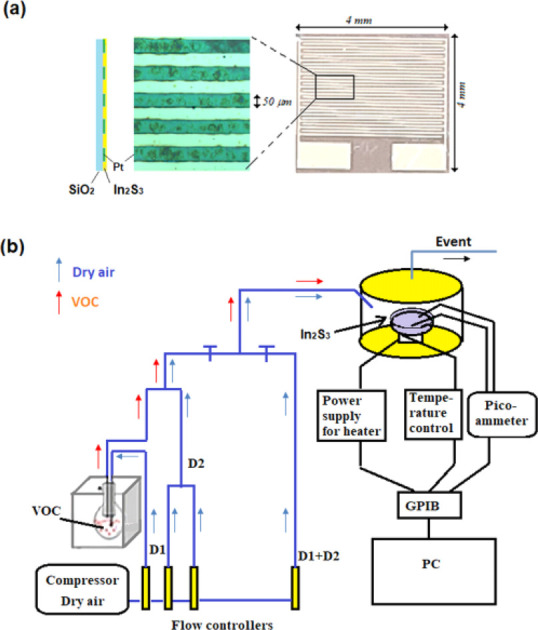

## 1. Introduction

Lung cancer is one of the deadliest diseases globally, with the highest morbidity and mortality rates among all cancers. Without early detection, it is nearly incurable, with a 5 year survival rate of only 15–20%.^1^ Early detection, however, can boost the survival rate to around 80%. Therefore, developing an easy and accurate early diagnosis method is crucial.

The presence of certain volatile organic compounds (VOCs) in human breath can indicate lung cancer.^2–4^ Using chemical sensors to detect these VOCs offers a non-invasive diagnostic approach. Hydrocarbons like ketones, alcohols, and aldehydes are considered cancer biomarkers.^5^ Among these, isopropanol levels are notably higher in lung cancer patients compared to healthy individuals and those with other lung diseases. Diabetic patients, for instance, have an average exhaled isopropanol concentration of 85.44 ppb.^6^ Breath analysis for alcohols is linear up to 420 ppb.^6^ Monitoring VOC concentrations can aid in early diagnosis, underscoring the need for a rigorous detection protocol.

Nanotechnology has advanced sensor sensitivity, selectivity, and stability through the development of new materials. In_2_S_3_ (indium sulfide) is a promising material for gas sensors due to its exceptional electrical properties and broad application range, including solar cells, gas sensors, and photocatalysts.^7–12^ While metal oxides are effective chemical gas sensors, they face selectivity challenges.^13,14^ Recent studies, including our own, have shown In_2_S_3_'s sensitivity to various gases like VOCs, O_3_, NO_2_, and NH_3_.^9,10,15,16^ Although cross-responses can affect selectivity, using hetero-structures or doping can enhance specific analyte detection,^17,18^ contributing to electronic nose applications.^19^

In this study, we propose addressing overlapping static responses by developing new features from the sensor's dynamic response. This will create a database for linear discriminant analysis to recognize isopropanol.

## 2. Experimental

**Fig. 1:**
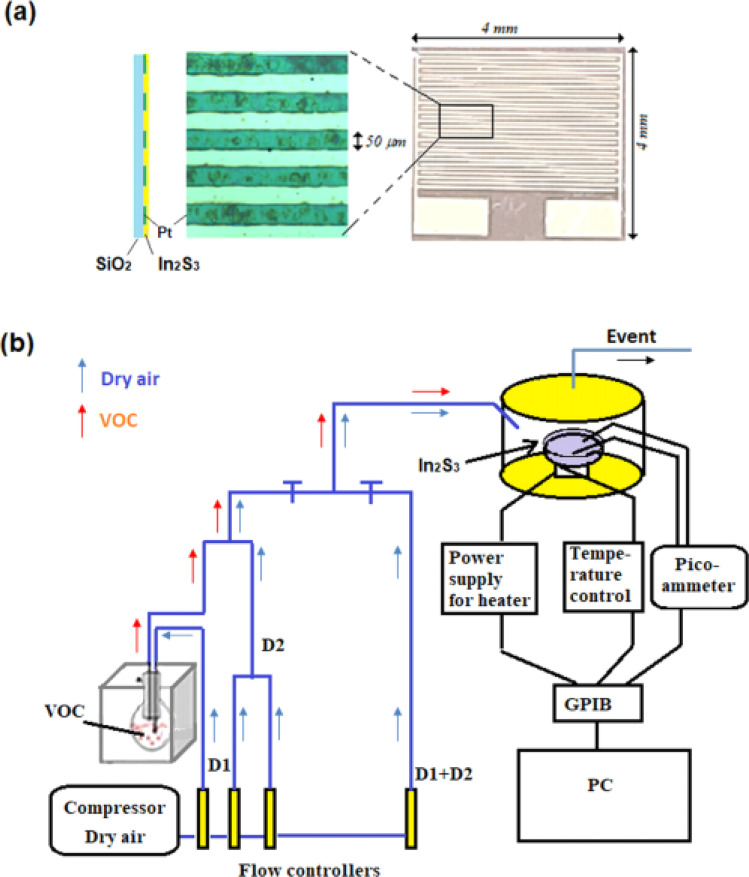
(a) Interdigitated microelectrodes array consisting of a total of 30 platinum fingers prepared by sputtering with photolithography and lift-up methods, where an indium sulfide film grows, (b) experimental setup for VOC detection.

Supplied from Sigma-Aldrich, thiourea, SC(NH_2_)_2_ (99.0%), and indium chloride InCl_3_ (99.0%) were added to distilled water to obtain initial solution. The latter was sprayed by pyrolysis for 180 s at 350 °C onto silicon substrates, equipped with interdigitated microelectrodes composed of a total of 30 platinum fingers (with an electrode spacing of 50 μm) prepared by sputtering with photolithography and lift-up methods on 4 × 4 mm^2^ surface as shown in Fig. 1(a). The last mentioned are previously immersed in an acetone bath for 15 min under ultrasound and then rinsed by distilled water and dried for 15 min. Spray pyrolysis is simple and inexpensive at reasonable temperatures (100 °C to 500 °C). It does not require high-quality targets or vacuum. The flow rates of solution and nitrogen gas were kept constant at 2 mL min^−1^ and 6 L min^−1^, respectively. The molar ratio of the S/In precursor solution was set to 2. The synthesis of In_2_S_3_ is given by the following equation.

1 2InCl_3_ + 3SC(NH_2_)_2_ + 6H_2_O → In_2_S_3_ + 3CO_2_ + 6NH_4_Cl

The layer thickness, *d* was estimated to be 200 nm using the double weight method and calculated by the following formula:2
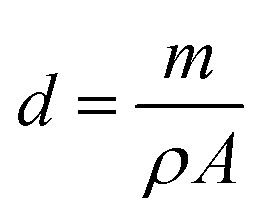
where *m* represents the mass of the deposited film, *ρ* is the bulk density of the material (with *ρ* = 4.613 g cm^−3^ consistent with the standard JCPDS file 25-390), and *A* is the effective area over which the film was deposited.

Given that the thickness of the interdigitated platinum electrode is 100 nm, and considering that adsorption occurs at the surface, it is reasonable for the film thickness to be slightly greater than that of the electrodes. This ensures a good electron flow during the gas sensing process.

Fig. 1(b) illustrates the experimental set-up employed for gas detection. In_2_S_3_ sensor is positioned on a support using two golden tungsten electrodes and polarized at 1 V utilizing an HP 4140B pico-ammeter source. The device is linked to a computer *via* a GPIB card for real-time recording electric current across the sensor. Heating of the sample is achieved by a halogen lamp. The working temperature ranges from 250 °C to 450 °C. Organic vapors are bubbling from a pure liquid contained in a round-bottomed flask positioned within a Polystat cc1 Huber water bath set at a fixed temperature (*T*_vap_). These vapors are subsequently diluted with dry air serving as the carrier gas. The concentration of VOC is regulated using a two-arm gas flow device. The first arm receives organic vapor (*D*_1_ flow rate), while the second arm is supplied with dry air (*D*_2_ flow rate). The two streams converge at the end of the two arms.

The VOC concentration is given by the following expression:^9^3
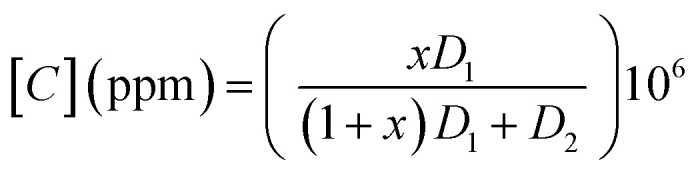
where 
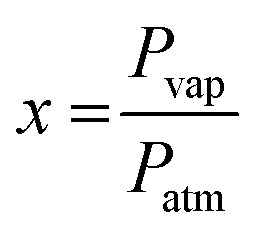
 is the molar ratio of VOCs at a predetermined *T*_vap_ temperature, *P*_vap_ is the partial pressure of volatile organic compounds at a given temperature *T*_vap_, and *P*_atm_ designates atmospheric pressure.

Following a 5 minute sensor exposure to VOCs, the test chamber will undergo a 15 minute cleaning with dry air, representing the typical duration needed to revert to the initial baseline level. The sensor response is defined accordingly:^9^4
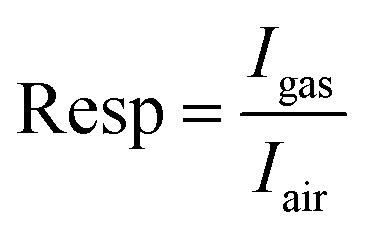
where *I*_gas_ is the current change caused by gas exposure and *I*_air_ designates to the baseline current in dry air.

Likewise, sensitivity is defined by eqn (5):^15^5
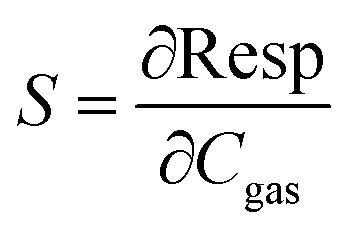
where *C*_gas_ designates the concentration of target gas. The sensitivity *S* of the tested sensors is represented by the slope of the fitting line correlating sensing response with concentration.

## 3. Results and discussion

### 3.1 Characterization

#### 3.1.1 Structural analysis

**Fig. 2:**
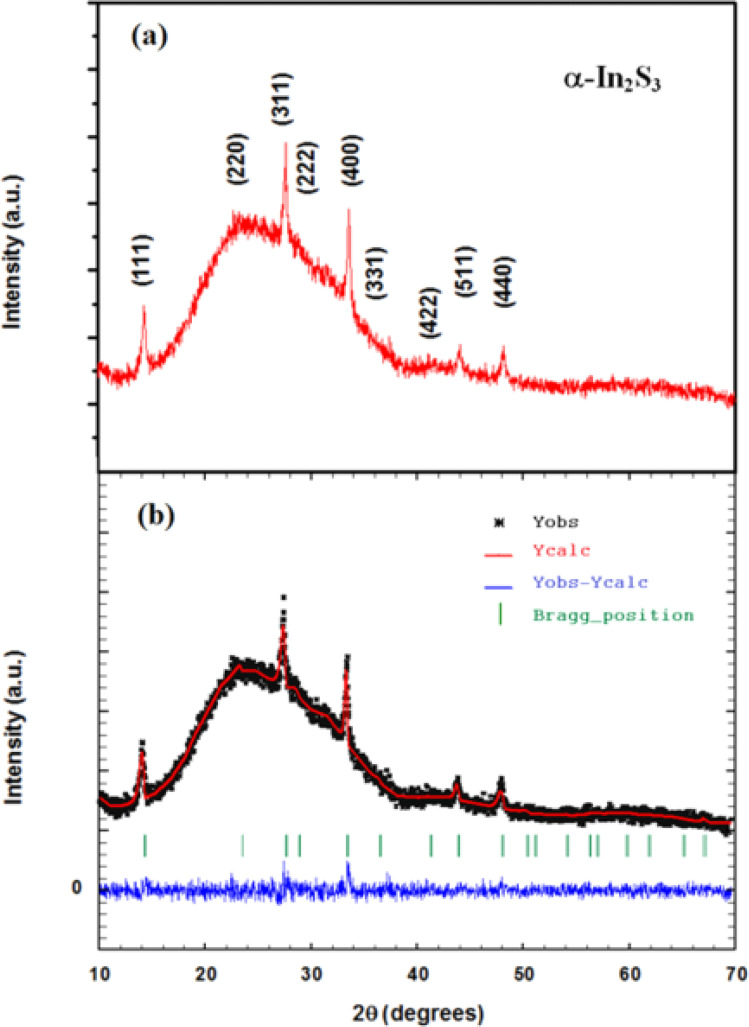
(a) X-ray diffraction patterns of α-In_2_S_3_ thin film, (b) observed patterns (black mark), Rietveld refinement (red line), gap between observed and Rietveld refinement patterns (blue line), Bragg positions (green).

**Fig. 3:**
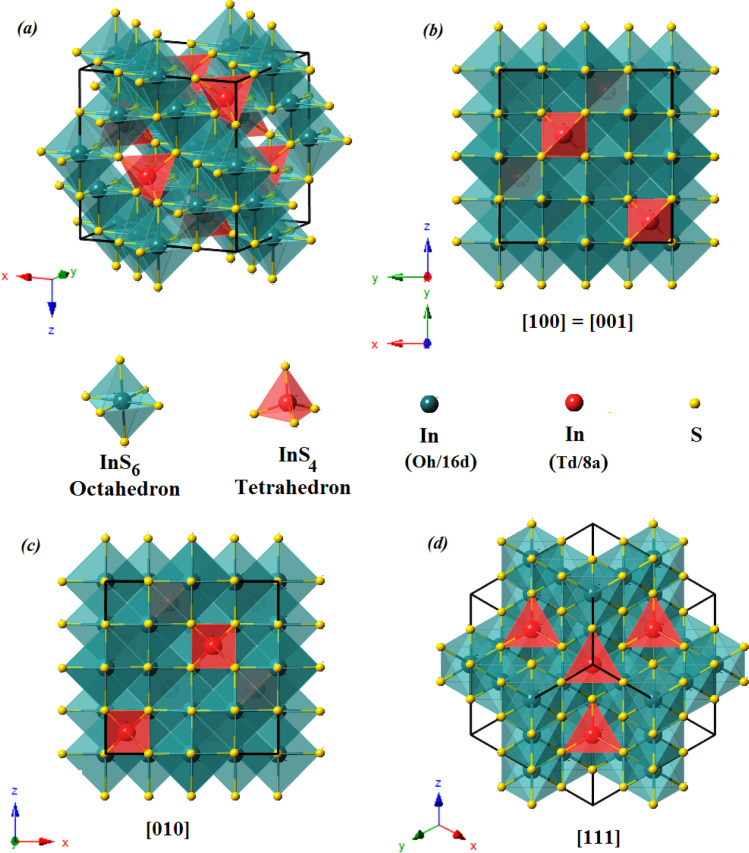
Schematic representation of the crystalline structure of the α-In_2_S_3_ thin film: (a) ground-state cubic structure (*Fd*3̄*m* space group), (b) projection along the [100] = [001] axis, (c) [010] axis, and (d) [111] axis.

X-ray diffraction patterns of In_2_S_3_ sprayed thin films are shown in Fig. 2(a). For the sake of clarity, we have included the Miller indices (*h k l*) of the most intense reflections of the *α* phase. The most notable Miller planes (1 1 1), (2 2 0), (3 1 1), (2 2 2), (4 0 0), (3 3 1), (4 2 2), (5 1 1) and (4 4 0) are located at around 2*θ* = 14.3°, 23.4°, 27.5°, 28.8°, 33.4°, 36.5°, 41.3°, 43.8° and 47.9° respectively. They are a typical diffraction pattern in the cubic α-In_2_S_3_ symmetry, and described in *Fd*3̄*m* space group according to the standard JCPDS card no. 32-0459.^20^ The Rietveld analysis plots, which were realized by FULLPROF software program^21^ of X-ray diffraction pattern at room temperature, show a good agreement between the observed and calculated patterns. The observed and calculated patterns are shown as the solid circles and the top solid line, respectively. The vertical markers stand for the angles of Bragg reflections. The lowest solid line corresponds to the difference between the calculated and observed intensities. To achieve the refinement, we have adopted Pseudo-Voigt peak profile function and approximate the background by a cubic spline interpolation between a set of background points with refinable heights, and taking into account the amorphous substrate contribution. Fig. 2(b) shows the refinement results of XRD patterns of In_2_S_3_ sprayed thin films. The structural parameters estimated from X-ray diffraction data are given in Table 1. The primitive unit cells of the structural models of the cubic α-In_2_S_3_ with a projection along the [100], [010], [001], and [111] axis is represented in Fig. 3 which takes a cubic spinel structure of space group *Fd*3̄*m*. Fractional occupation of In atoms occurs in tetrahedrally coordinated in Td(8*a*) sites at the Wyckoff position (8*a*) (1/8, 1/8, 1/8), and octahedrally coordinated *O*_h_(16*d*)-sites at the Wyckoff position 16*d* (1/2, 1/2, 1/2), and S ions at (32*e*) sites (*x*, *x*, *x*). The resulting higher crystal symmetry explains the observed disappearance of some of the minor intensity peaks in the diffractograms compared to another indium sulfide.^22^ This is in good agreement with the results in a previous report.^20,23–25^ Various fitting parameters are used to assess the quality of fitting simulated data on the experimental plot. Reliability index parameters in form of *R*_p_, *R*_wp_, *R*_exp_ has been estimated. *R*_p_ is the residual error, *R*_wp_ is the weighted residual error, *R*_exp_ is the expected residual error. The obtained reliability factors, the goodness-of-fitting (*χ*^2^), and lattice parameters calculated from the Rietveld refinements are tabulated in Table 1.

Structural parameters for the cubic α-In_2_S_3_ (space group 227)

**Table:** 

Synopsis	
Formula	α-In_2_S_3_
Crystal system	Cubic
Space group	*Fd*3̄*m*
Density (g cm^−3^)	4.686

**Table:** 

Lattice parameters	
*a* = *b* = *c* (Å)	10.7189 (8)
*α* = *β* = *γ* (°)	90
Volume, *V* (Å^3^)	1231.55 (9)

**Table:** 

Atomic coordinates					
Atoms	Wyckoff	*x*	*y*	*z*	Occ.
In	8*a*	0.1250	0.1250	0.1250	0.34(2)
In	16*d*	0.5000	0.5000	0.5000	1.66(2)
S	32*e*	0.2524(9)	0.2524(9)	0.2524(9)	3

**Table:** 

Reliability index parameters			
*R*_p_ (%) = 3.32	*R*_wp_ (%) = 4.25	*R*_exp_ (%) = 4.11	*χ* ^2^ = 1.07

#### 3.1.2 Surface analysis

**Fig. 4:**
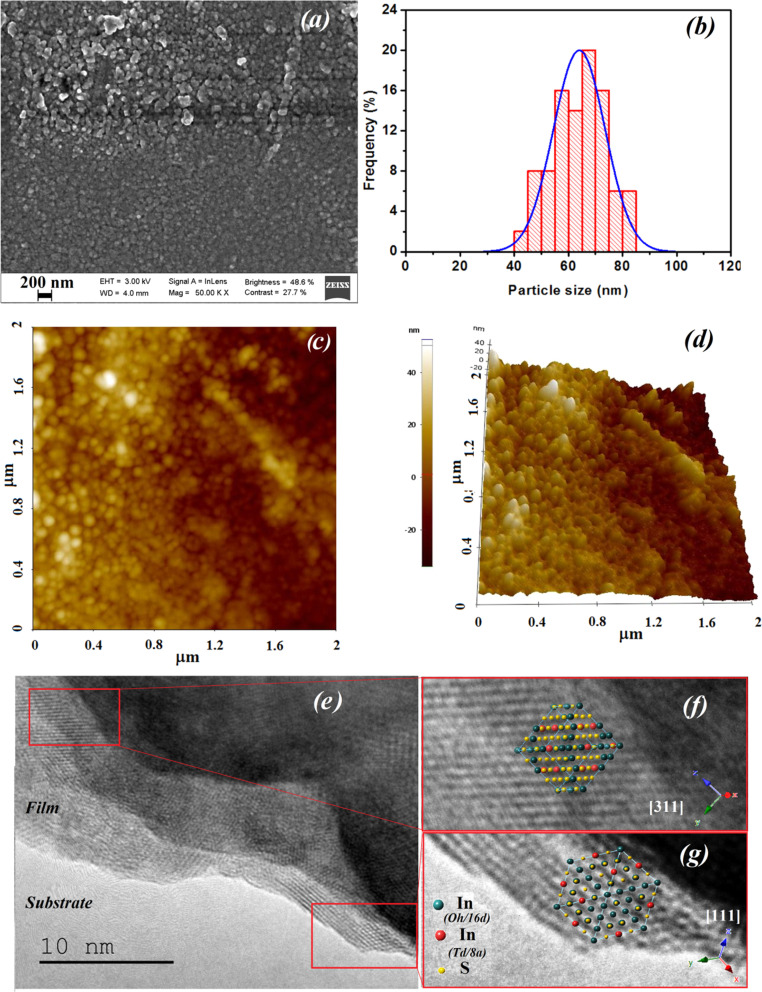
(a) SEM image of the In_2_S_3_ film, (b) histogram for the grain size distribution measured from SEM image, (c) 2D AFM image, (d) 3D AFM image, (e) TEM image of In_2_S_3_ grain, (f) schematic reconstruction of atomic sites of [311] imaged orientation axis for the left enlarged red squared area of sub-figure (e), (g) schematic reconstruction of atomic sites of [111] imaged orientation axis for the right enlarged red squared area of sub-figure (e).

SEM investigation reveals that surface of α-In_2_S_3_ thin film is fairly covered by the coalescence of surrounding crystallites, resulting in rather large aggregates in 3D growth state (Fig. 4(a)). It is evident that the particle size (nearly spherical structure) is in the range of 40–85 nm Fig. 4(b). This finding confirms that the α-In_2_S_3_ particles are a dense cluster of tiny grains. AFM analysis produced comparable findings. Typical 3D and 2D AFM pictures in a region of 2 μm × 2 μm are shown in Fig. 4(c) and (d). The three-dimensional atomic force microscopy was used to understand the orientation of the grains and also to estimate the root mean square (RMS) surface roughness. At the left side of AFM images, an intensity strip is shown, which shows the depth and the height along the *z*-axis. AFM observations show a disturbed surface with random oriented crystallites showing a rough surface with an RMS surface roughness around 13 nm. This suggests that the growth mechanism of α-In_2_S_3_ is of two types: ion-by-ion and cluster-by-cluster. This is in correlation with the change of the preferred orientation observed by XRD analysis. Fig. 4(e)–(g) displays TEM images of a grain of the thin film with two areas identified as oriented along [111] and [311] axis. TEM image supported the presence of several domains with different orientations, this observation confirms that the α-In_2_S_3_ particles are in fact compact agglomeration of small grains in polycrystalline state.

These checked characterizations are crucial for ensuring that the material provides a sensitive surface with nanoparticles sized between 40 and 85 nm, offering an excellent sensing area with a roughness of 13 nm. Additionally, they help us to optimize metrological parameters for future studies. Each manufacturing or deposition parameter can significantly impact the sensor's performance.

#### 3.1.3 Impedance spectroscopic analysis

**Fig. 5:**
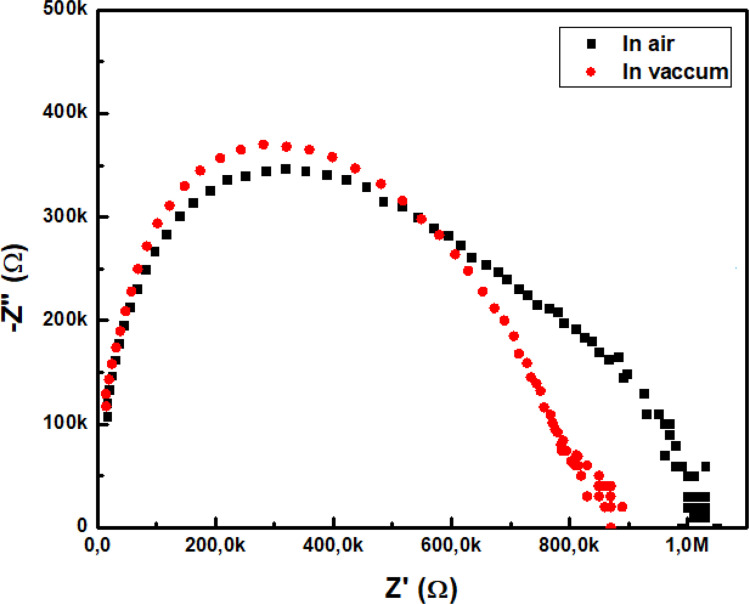
Complex impedance plots of In_2_S_3_ at 300 K, measured in both vacuum and air.

AC electrical measurements were performed using an impedance analyzer (HP LF 4192A). Initially, the film was placed in a vacuum test chamber and the pressure was reduced to 10^−3^ torr. Subsequently, air was introduced into the chamber. The impedance behavior of In_2_S_3_ nanoparticles at 300 K was examined across a frequency range from 0.1 Hz to 100 kHz. Fig. 5 illustrates the relationship between the real (*Z*′) and imaginary (*Z*′′) components of the complex impedance, forming two distinct arcs.

In_2_S_3_ is an n-type wide band-gap semiconductor characterized by sulfur vacancies that act as donors.^26,27^ When the film is exposed to air, molecular oxygen accepts electrons, reducing the electron concentration in the conduction band. Oxygen, in forms such as O_2_^−^, O^−^, or O^2−^ depending on temperature, is chemisorbed on the surface and at grain boundaries. This chemisorption creates a surface Schottky barrier and an electron-depleted region, increasing the material's resistance.^28^ During the pumping phase, the film releases adsorbed oxygen as molecular O_2_. This desorption frees one or two electrons per atom, which re-enter the conduction band. This process lowers the resistance by reducing the potential barrier at the grain boundaries and increasing the carrier density.

### 3.2 Isopropanol sensing properties

#### 3.2.1 Isopropanol sensing mechanism

**Fig. 6:**
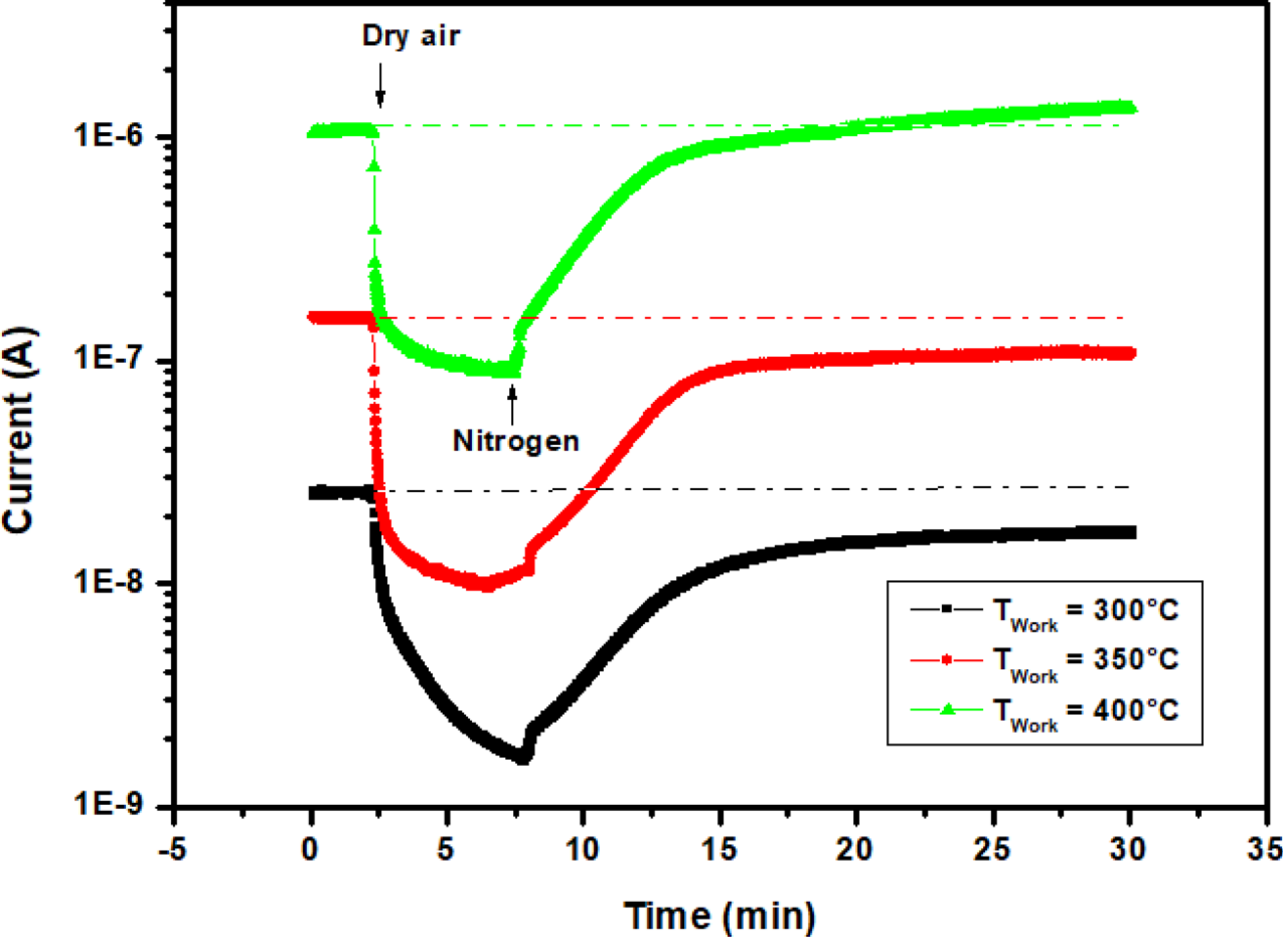
Current change through In_2_S_3_ film *vs.* time upon altering exposure to dry air and nitrogen at operating temperatures 300 °C, 350 °C and 400 °C.

Before performing isopropanol sensing tests, the oxygen adsorption and desorption behavior on In_2_S_3_ sample was experimentally explored a second time. This was done by exposing the film to nitrogen and dry air alternately at temperatures of 300 °C, 350 °C, and 400 °C. As shown in Fig. 6, the current decreases when dry air is injected into the test chamber and increases when it is purged by nitrogen. This behavior is related to the adsorption/desorption phenomenon of oxidizing gas on the surface revealing the n-type of In_2_S_3_ semiconductor and confirming the results obtained in the previous section. Indeed, in dry air, oxygen molecules adhere to the surface of the In_2_S_3_ film. These adsorbed oxygen molecules then capture electrons from In_2_S_3_ nanoparticles due to the strong electronegativity of oxygen, generating ionized oxygen species such as O_2_^−^, O^−^, and O^2−^ which decreases the current through the sensor.^28,29^

**Fig. 7:**
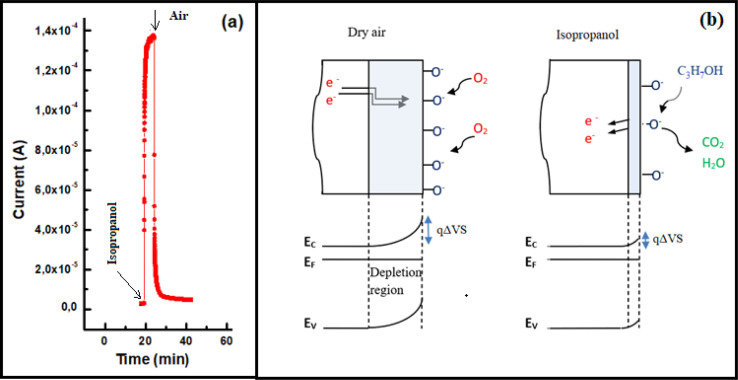
(a) Dynamic change of current during a cycle of alternating exposure of In_2_S_3_ film to 500 ppm isopropanol and dry air at 350 °C, (b) schematic isopropanol sensing mechanism.

Fig. 7(a) illustrates the transient current variation of an In_2_S_3_ film when alternating between 500 ppm isopropanol and dry air within the test chamber at 350 °C. As depicted, the sample exhibits an increase in current when exposed to isopropanol and a return to baseline level when subjected to dry air. This observed behavior aligns with the detection mechanism of reducing gases by n-type semiconductors.^30,31^

Upon isopropanol vapors injection into the test chamber, the molecules react with the previously adsorbed oxygen species and electrons were released to In_2_S_3_ layer. In previous work^32^ we demonstrated that O^2−^ ions in the layer interact mainly with VOCs. Hence, the isopropanol sensing mechanism in indium sulfide film can be elucidated by the following equations:

Under dry air:6
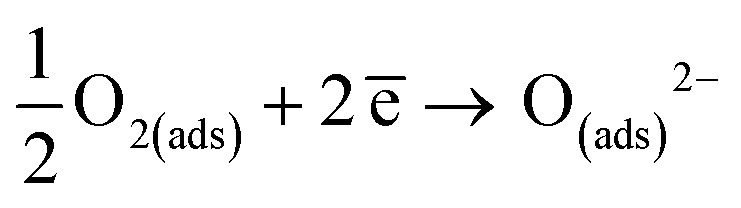


Under isopropanol:

7 C_3_H_7_OH_(ads)_ + 9O_(ads)_^2−^ → 3CO_2_ + 4H_2_O + 18ē

eqn (6) illustrates that the adsorption of oxygen on the In_2_S_3_ surface effectively captures electrons. Since the material is of n type, this process reduces the Fermi level, leading to band bending and widening of the electron depletion region (refer to Fig. 5(b)). When isopropanol is introduced into the test chamber, the release of electrons according to the reaction mechanism (eqn (6)) increases the concentration of free carriers, thus raising the current intensity. Thus, the gas sensing mechanism is contingent upon the modification of potential barriers (qΔVS) between In_2_S_3_ grains due to the adsorption/desorption of oxygen ions, altering the surface electron depletion region as depicted in Fig. 7(b)

#### 3.2.2 Response

**Fig. 8:**
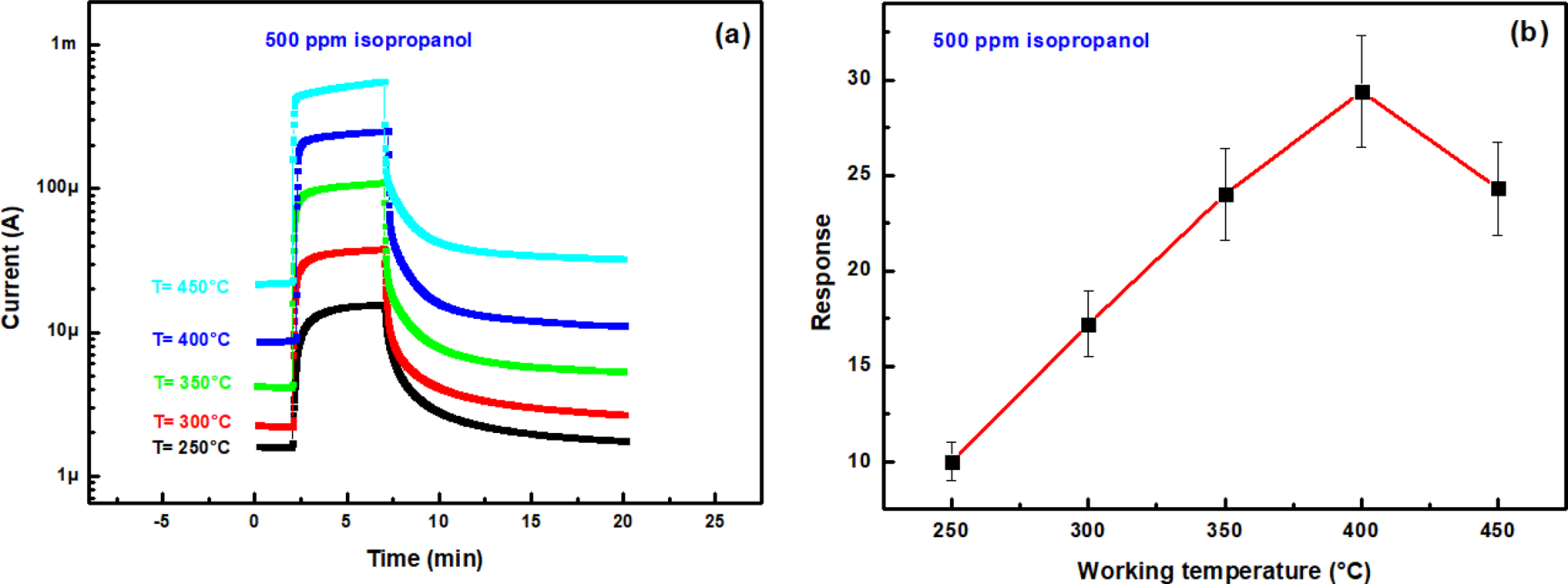
(a) Current changes of In_2_S_3_ film upon exposure to 500 ppm isopropanol at different operating temperatures (250 °C, 300 °C, 350 °C, 400 °C and 450 °C), (b) sensing response *vs.* working temperature.

In order to check the response of the prepared sample to isopropanol vapor, sensing tests were performed under dry air flow, in the temperature range 250–450 °C. Fig. 8(a) shows current change through In_2_S_3_ film upon exposure to 500 ppm isopropanol at different operating temperatures and Fig. 8(b) reports the dependence of the sensor response on temperature. Up to 200 °C, the sensor response is low, indicating that activation of the isopropanol reaction with oxygen adsorbed on the surface requires higher temperatures. As the temperature approaches 250 °C, the response increases. The sample has better performance at high temperature and the optimal operating temperature is 400 °C showing a maximum response of 28. In fact, this phenomenon was intricately linked to the varying adsorption capacities of gas molecules on the sensing material surface at different temperatures. Besides, increasing the operating temperature favors the formation of adsorbed oxygen species on the surface of the sensing layer which enhances the oxidation–reduction reactions involving chemisorbed oxygen and isopropanol.

#### 3.2.3 Sensitivity

**Fig. 9:**
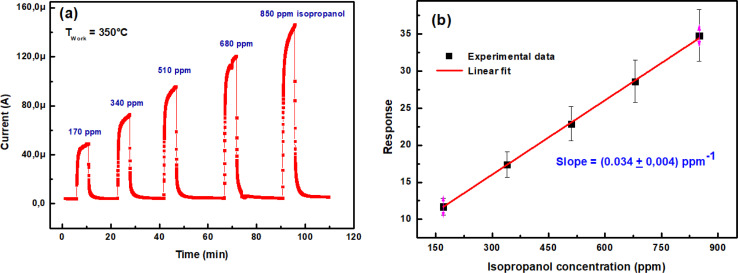
(a) Transient current intensity *vs.* time upon exposure of In_2_S_3_ sensor to various isopropanol concentrations at 350 °C, (b) sensing response *vs.* isopropanol concentration.

Fig. 9(a) represents current change of the In_2_S_3_ gas sensor to isopropanol input with different concentrations operated at 350 °C. One can see that the current increases upon exposure to isopropanol and the reached steady state current changes of 11.7, 17.4, 22.9, 28.6 and 34.8 times with respect to the baseline are observed towards 170, 340, 510, 680 and 850 ppm isopropanol, respectively.

The response as a function of gas concentration is plotted in Fig. 9(b). The sensing response is linearly proportional to isopropanol concentration in the range 150–900 ppm. The sensitivity of this sensor is calculated from the response slope of the fitted line and reaches (0.034 ± 0.004) ppm^−1^. This last value allowed to deduce the limit of detection (LOD) which is defined as the lowest concentration where the signal is distinctly distinguishable from the background noise (usually LOD is set at three times the standard deviation of the noise). To estimate sensor noise, the sensing response of the sensor is measured in its baseline state before exposure to volatile organic compounds (VOCs). This involves obtaining the average sensing response (Resp) from a series of baseline measurements (*N* = 30), followed by calculating the root mean square deviation (RMSD) using the formula provided^33,34^8
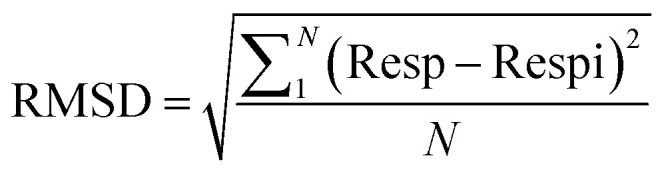


*N* represents the number of data points and Respi denotes the *i*th baseline measurement response, the (LOD) was computed using eqn (9) as per this definition.9
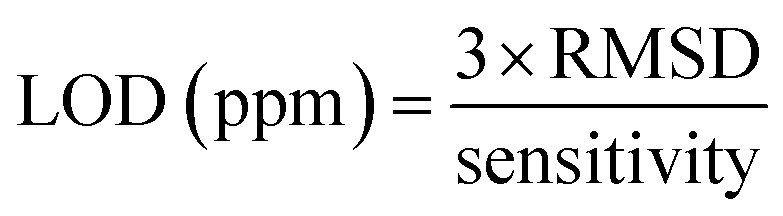


The computed LOD value is (162 ± 19) ppb. This value is sufficient to explore the detection of isopropanol as a biomarker in human breath according to recognized standards.

#### 3.2.4 Rapidity

The evaluation of a gas sensor's performance relies heavily on a crucial metrological parameter known as rapidity. This parameter can be dissected into response and recovery times (*τ*_rep_ and *τ*_rec_, respectively). The response time signifies the duration necessary for the sensor to achieve 90% of its response, while the recovery time indicates the duration needed to return to 10% of the initial resistance baseline after purging the analyte gas.^35^ The extracted values from 170 ppm isopropanol response at 350 °C of response time and recovery time are respectively 113 s and 42 s.

#### 3.2.5 Reproducibility and stability

**Fig. 10:**
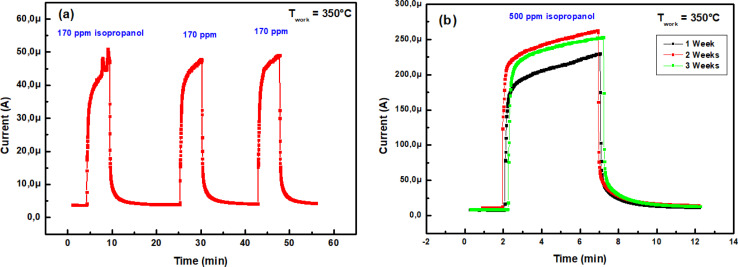
(a) Reproducibility of the In_2_S_3_ sensor at 350 °C towards 170 ppm isopropanol vapor, (b) stability at 350 °C towards 500 ppm during three weeks.

Both reproducibility and stability must also be met. Three consecutive cycles of exposure to 170 ppm isopropanol at 350 °C showed no significant change in sensing response (Fig. 10(a)). This consistent result suggests the sensor's good repeatability regarding isopropanol. Similarly, the long-term stability of a gas sensor indicates its capacity to maintain a steady response to the same gas concentration over an extended period. Fig. 10(b) underscores the stability of our material over three weeks when exposed to 500 ppm isopropanol at 350 °C.

The performance metrics of this new type of In_2_S_3_-based isopropanol sensing material prepared by simple spray pyrolysis were compared with other similar works published in the literature shown in Table 2. These results reveal that the best detection limit is found in this work. Moreover, the sensing properties of the In_2_S_3_ sensor are encouraging to further study to enhance the selectivity of the sensor as detailed in the following text.

**Table:** Comparison of isopropanol sensing performance metrics of In_2_S_3_ with other metal oxide-based sensors in the literature

Material	*T* (°C)	Concentration (ppm)	Response	*τ*_resp_/*τ*_rec_ (s)	LOD (ppb)	Year	Ref.
NiO/NiCo_*x*_Fe_2−*x*_O_4_	183.5	100	11.2^a^	—/—	—	2023	36
rGO-ZnO nanofibers	225	50	23.2^a^	14/39		2023	37
Fe-doped ZnO nanoneedles	275	5	23.6^a^	51/762	250	2021	38
ErFeO_3_	270	100	21^b^	6/25	2000	2021	39
ZnMn_2_O_4_ nanoparticles	250	100	0.99^c^	23/70	666	2021	40
ZnSnO_3_ nanospheres	200	10	10.3^b^		500	2020	41
Double-shelled SnO_2_ cubes	180	100	14.5^a^	1/33	—	2019	42
In_2_S_3_ nanoparticles	350	170	11.7^a^	113/42	162	2024	This work

a Response = *R*_air_/*R*_gas_.

b Response = *R*_gas_/*R*_air_.

c Response = (*R*_air_ − *R*_gas_)/*R*_air_.

#### 3.2.6 Selectivity

**Fig. 11:**
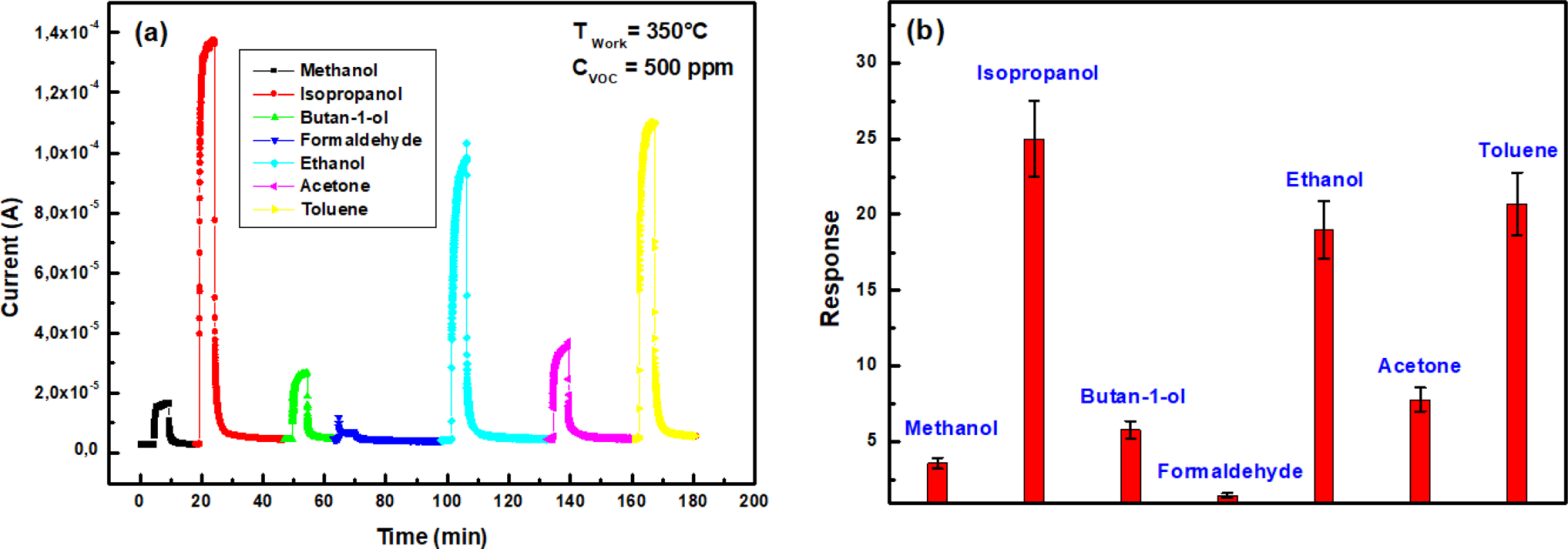
(a) Transient current change of In_2_S_3_ sensor at 350 °C towards different VOCs for 500 ppm concentration, (b) sensing response to 500 ppm VOC at 350 °C.

Selectivity, a crucial sensor characteristic, refers to its ability to detect a specific gas amidst the presence of other gases. This property is typically assessed at a steady state by comparing the sensor's response to the target gas with that of an interfering gas, both at equal concentrations. Fig. 11(a) illustrates the change in current over time for the In_2_S_3_ film when exposed to seven different VOCs at a concentration of 500 ppm and a temperature of 350 °C. In Fig. 11(b), notable responses are recorded for isopropanol (25), toluene (20.7), and ethanol (19), indicating relatively high reactivity. Acetone (7.8) and butan-1-ol (5.8) exhibit intermediate responses, while methanol (3.6) and formaldehyde (1.5) vapors elicit weaker reactions. This occurrence was closely linked to the adsorption capacity of gas molecules on the surface of the sensing material, as well as the oxidation–reduction reactions involving chemisorbed oxygen.^43,44^

We note a cross-responses between isopropanol, ethanol and toluene as shown in Fig. 11(b). Despite the higher response to isopropanol there was poor selectivity. Hence, at present, In_2_S_3_ sensor's selectivity cannot be assured. In order to solve this problem and achieve isopropanol recognition, we came up with the idea of applying linear discriminant analysis (LDA) to features arising from transient current change measurements. Indeed, LDA is a graphical data analysis method aimed at identifying the most representative trends in space that capture the interconnections among the selected measurement variables.^45^

### 3.3 Linear discriminant analysis for real time selectivity

Linear Discriminant Analysis (LDA) is a widely-used and straightforward statistical algorithm.^46,47^ It operates by creating vectors of linear combinations of features associated with individuals, aiming to maximize the variance between different classes while minimizing the variance within the same class. The data matrix for LDA consists of rows representing individual names and columns corresponding to their respective features. LDA works with a static database and is categorized as a supervised machine learning. In our study, we focused on examining the transient response of the sensor during the injection of target steam. We have identified and extracted key features from this system, which will be discussed in detail in the following section.

#### 3.3.1 Features emerged from the transient responses

**Fig. 12:**
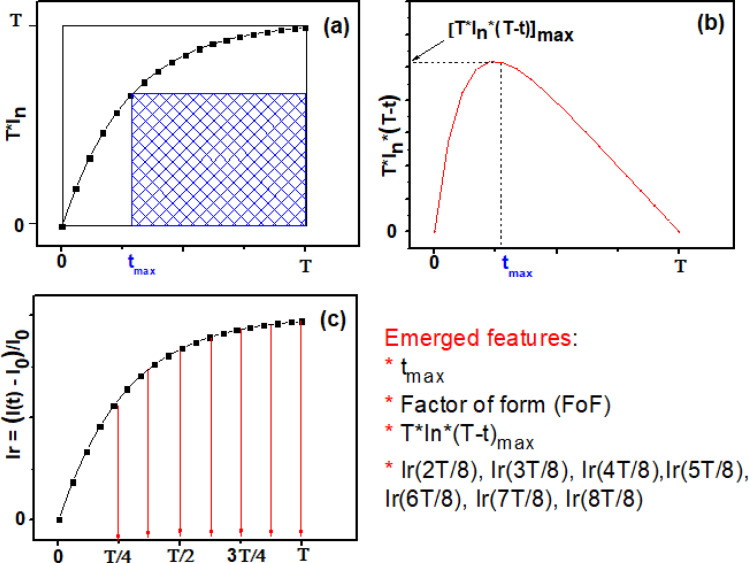
A model of the emerged features derived from the transient current, (a) graph of normalized and squared current (*T* × *I*_n_) evolution, the blue shaded area represents the FoF numerator, (b) appearance of [*T* × *I*_n_ × (*T* − *t*)] function generated from curve (a), (c) relative dynamic response at 2*T*/8, 3*T*/8, 4*T*/8, 5*T*/8, 6*T*/8, 7*T*/8 and 8*T*/8.

In prior research,^48^ we established computer code aimed at incorporating novel aspects of the sensor's real-time transient response. Within this section, our focus lies solely on the reactions observed at 350 °C for all analyzed VOCs. Our initial procedure involves standardizing the transient sensing response across the duration *T* (where *T* equals 1 minute (Fig. 12(a))) using the subsequent formula:10
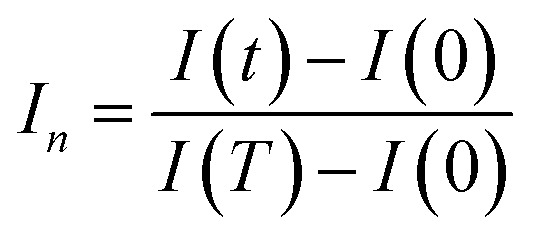
where *I*(*t*) the current under VOC at the instant *t* and *I*(0) is the current in dry air just before VOC injection.

Fig. 12(a) illustrates the time-dependent normalized and squared response, *T*·*I*_n_, within the interval [0, *T*]. Furthermore, Fig. 12(b) depicts a distinct curve derived from the normalized response using the subsequent expression:

11 *P*(*t*) = [*T*·*I*_n_(*t*)·(*T* − *t*)]

It is observed that *P*(*t*) reaches its peak at a specific time instant, denoted as *t*_max_, which can indirectly reflect the kinetics of the chemical reaction of the target gas on the surface. The value of *t*_max_ is correlated with the normalized and squared response depicted in Fig. 12(a), as it signifies the point crucial for determining the factor of form (FoF), as defined by the ensuing formula:12
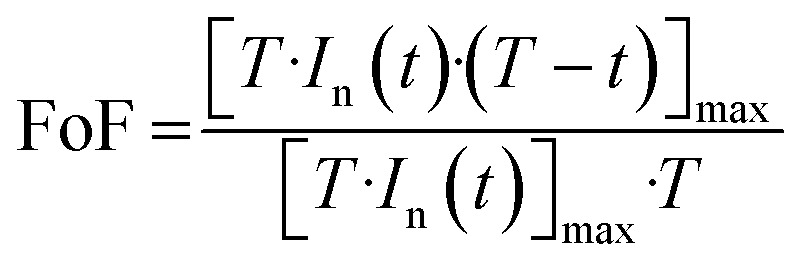


Visually, the FoF is represented by the ratio of the blue shaded area to the total square area (*T*^2^), as illustrated in Fig. 12(a).

In addition, the relative response *I*_r_(*t*) is expressed as follow:13
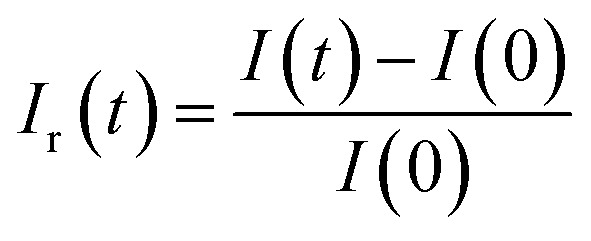


Subsequently, through the execution of the computer code, we adeptly extract 10 features, specifically: *t*_max_, FoF, [*T*·*I*_n_·(*T* − *t*)]_max_, the relative responses *I*_r_(*t*) relating at instants (15 s, 22.5 s, 30 s, 37.5 s, 45 s, 52.5 s, 60 s).

This enabled us to construct a labeled database for conducting linear discriminant analysis.

#### 3.3.2 LDA methodology and results

LDA is a dimensionality reduction technique commonly employed as a preprocessing step in sample classification and machine learning applications.^45^ At its core, LDA aims to enhance the distinction between two clusters. The objective is to map a feature space (comprising *n*-dimensional data) onto a smaller subspace (where dimensional data is less than *n*), while retaining the class-discriminative information. This can be achieved by directly optimizing the linear decision boundary, without relying on any probabilistic model, and can be approached in several ways. This method seeks a vector W such that:

• It maximizes the separation between the centroids of each cluster projected onto this line, thereby increasing the between-clusters distance.

• It minimizes the distance of observations from their respective centroids, thereby reducing within-cluster variance.

The classical LDA method is extensively utilized across various applications in a conventional manner, typically interpreted through a standard subspace discrimination algorithm for binary classification problems. By assembling a dataset derived from a vector matrix comprising two classes, we can calculate the between-class scatter matrix *S*_B_ and the within-class scatter matrix *S*_W_ using eqn (14) and (18) respectively.^45–50^14
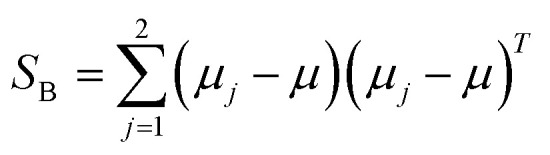
where *μ*_*j*_ denotes the centroid of all samples of class *j* defined by the following equation:15
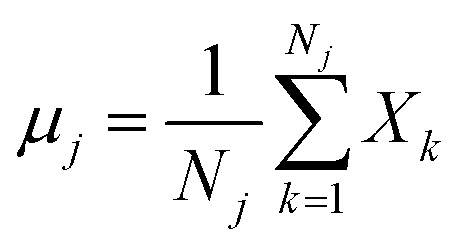
16
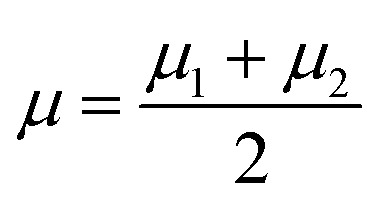
in order to compute the within-cluster matrix *S*_W_, we calculate the matrix *S*_*j*_ corresponding to each cluster *j*.17
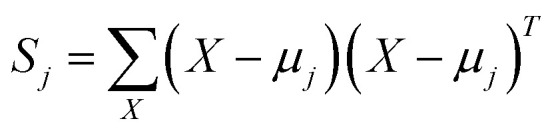
18
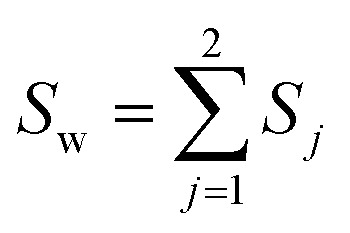


The objective in LDA is to determine the optimal projection vector *W*. This vector *W*, responsible for space transformation, is obtained for dimensionality reduction by solving the following Rayleigh quotient:^45–50^19
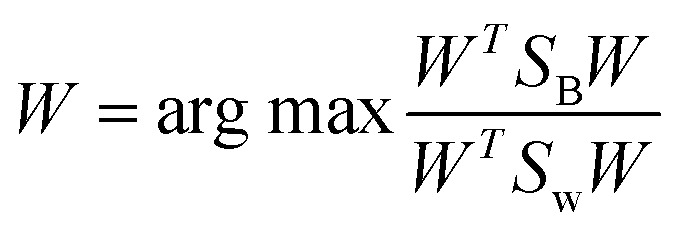
in this context, *W* represents the eigenvector associated with the largest eigenvalue, determined using specific eqn (20) and (21):

20 *S*_W_^−1^*S*_B_*V* = *λV*

21 |*S*_W_^−1^*S*_B_ − *λI*| = 0

alternatively, we can directly solve for the W vector using the following formula:^45^

22 *W* = *S*_W_^−1^(*μ*_1_ − *μ*_2_)

Supervised analysis involves integrating the learned features of VOCs with the database to confirm their belonging, thus determining their classification into one of two classes. Therefore, the outputs are binary, represented as either 0 or 1. This enables us to choose isopropanol for its separation from the other VOCs. Subsequently, we establish the projection vector that constitutes the learning outcome. To achieve this, we utilized a matrix comprising 11 measurements, encompassing five concentrations ranging from 170 to 850 ppm for isopropanol and a single concentration of 500 ppm for each remaining volatile compound (ethanol, methanol, acetone, formaldehyde, butan-1-ol and toluene). Each measurement correlates with ten features extracted from the transient response occurring before reaching a steady state (0 < *t* < *T* = 1 min). Initially, we partitioned our supervised measurements into two groups: the first group comprised solely of isopropanol, while the second group encompassed the remaining six VOCs.

**Fig. 13:**
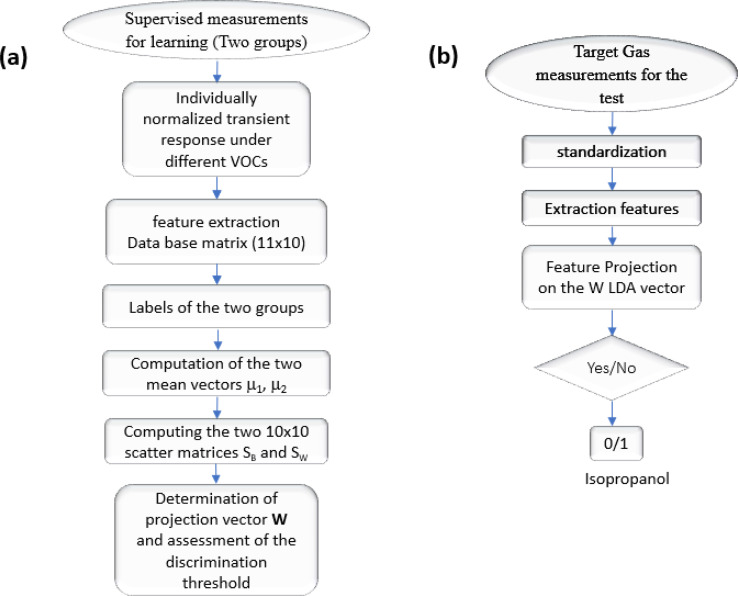
(a) Flowchart showing the steps of automatically pick out isopropanol molecules, (b) test procedure for the isopropanol target.

The process of automating the Linear Discriminant Analysis (LDA) algorithm is outlined in the flowchart depicted in Fig. 13(a) and (b), which illustrates all the computational steps involved and the test procedure for the isopropanol target. Given that LDA is a supervised technique, individual test data cannot be incrementally added to the existing database. Additionally, our approach is constrained to classifying into two distinct groups, resulting in a binary outcome (yes/no). For positive classifications, we can reference the isopropanol concentration using the calibration curve provided in Fig. 9(b).

**Fig. 14:**
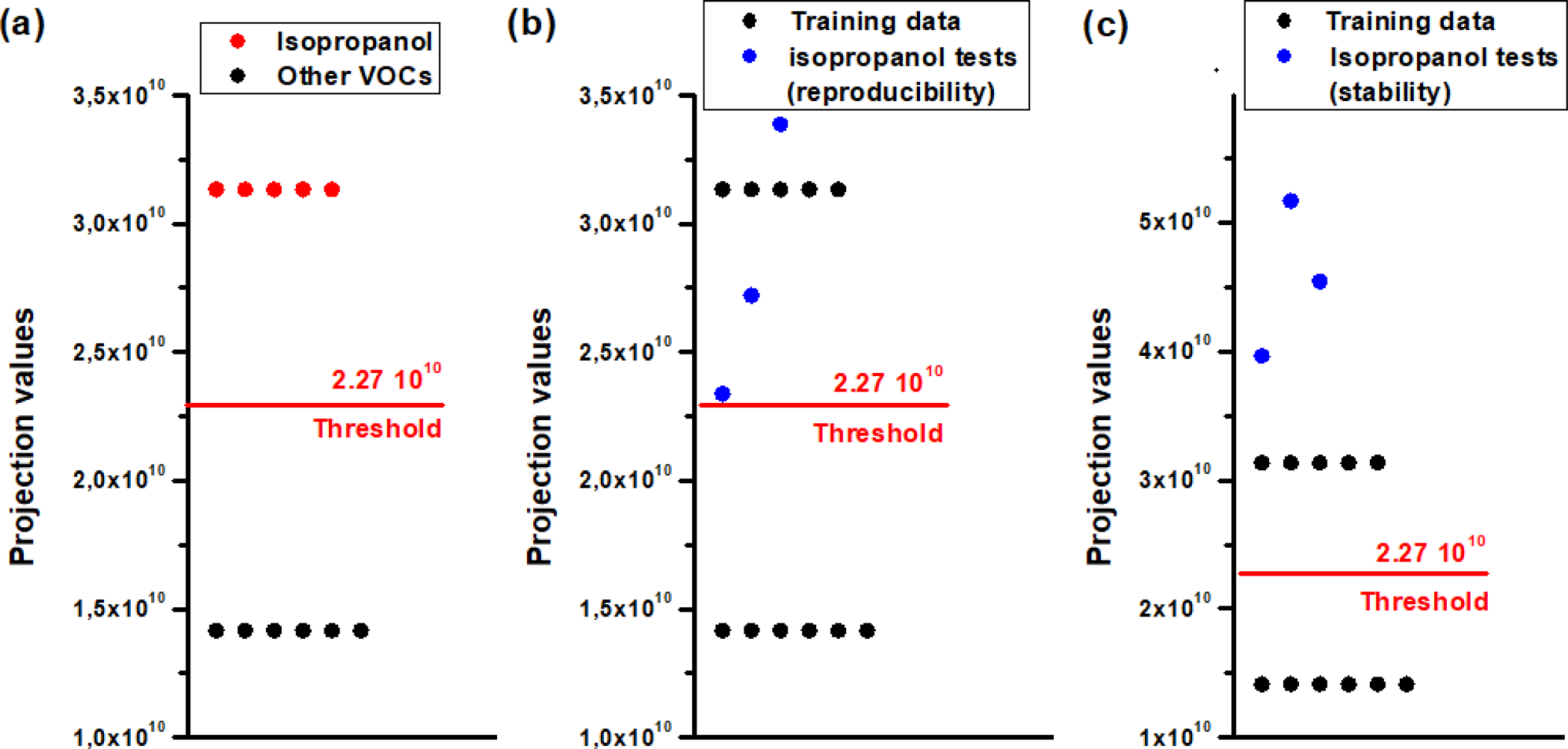
LDA outputs for identifying isopropanol vapor (a) training data, (b) isopropanol reproducibility test, (c) isopropanol stability test.

The outcome was remarkably conclusive, as depicted in Fig. 14(a), enabling us to establish a separation threshold of 2.27 × 10^10^ along the LDA projection axis for these two groups.

To authenticate our separation method, we conducted numerous tests involving isopropanol. We have processed the measurements depicted in Fig. 10(a) and (b) using code to extract the transient features described in the preceding paragraph. These features were subsequently projected onto the axis defined by the vector *W* (eqn (22)), which was derived from the learning outputs through the LDA method. Fig. 14(b) and (c) illustrates the LDA outcomes for the repeatability test conducted over three consecutive cycles and the stability test spanning three weeks, respectively. It is noteworthy that the detected isopropanol vapor aligns closely with its designated group in both tests.

Practical implementation can be done by volunteers who participate by providing breath samples in accordance with the biomedical research ethics protocol. Each volunteer should breathe deeply into the collection bag using the mouthpiece, making sure to do so before eating or drinking and after rinsing their mouth with clean water. The bag must be equipped with a tight check valve to ensure that only exhaled air flows into the bag and to prevent any outside air from entering. Breath samples will be collected in triplicate and immediately transferred to the sensor networks by pumping the contents of each bag for 5 minutes at a specified rate.

## 4. Conclusion

This work is focused on the integration of In_2_S_3_ material as an isopropanol sensor. The latter is relatively inexpensive compared to other advanced materials commonly used in sensor technology. Its cost-effectiveness, combined with its promising performance characteristics, makes it an attractive option for large-scale implementation in isopropanol detection for lung cancer diagnostics. Using the spray technique, we deposited In_2_S_3_ layers on SiO_2_/Si substrates with inter-digitized platinum electrodes. The refinement of XRD patterns for In_2_S_3_ films using the Pseudo-Voigt method demonstrates a strong agreement between calculated and observed intensities. The lattice parameters for cubic α-In_2_S_3_ were determined as *a* = *b* = *c* = 10.7189 Å, with the space group identified as *Fd*3̄*m*. SEM analysis indicates that the surface of the α-In_2_S_3_ thin film is relatively uniform, with crystallites coalescing to cover the surface. The particle size distribution falls within the range of 40–85 nm. AFM observations reveal a disrupted surface morphology characterized by randomly oriented crystallites, resulting in a rough surface with an RMS surface roughness measuring approximately 13 nm. The isopropanol sensing response *versus* working temperature shows an optimal temperature around 400 °C. The response of the material increases from 12 to 34 by varying isopropanol concentration from 170 to 850 ppm at 350 °C. The sensitivity was found to be 0.034 ppm^−1^. The sensor reveals good reproducibility and stability. Selectivity was strengthened *via* LDA mathematic tool by introducing genius features from transient response. The result ensures very good recognition of isopropanol.

## Data availability

The datasets generated during and/or analyzed during the current study are available from corresponding author on reasonable request.

## Author contributions

Conceptualization: R. Souissi, B. Bouricha. Data curation: R. Souissi, B. Bouricha. Formal analysis: R. Souissi, B. Bouricha, N. Ihzaz. Methodology: R. Souissi, B. Bouricha. Validation: R. Souissi, B. Bouricha, N. Ihzaz, N. Bouguila, M. Abderrabba. Writing – original draft: R. Souissi, B. Bouricha, N. Ihzaz. Writing – review & editing: N. Bouguila, M. Abderrabba.

## Conflicts of interest

There are no conflicts to declare.
